# A Review of Polymer Composites Based on Carbon Fillers for Thermal Management Applications: Design, Preparation, and Properties

**DOI:** 10.3390/polym13081312

**Published:** 2021-04-16

**Authors:** Yeon-Ju Kwon, Jung-Bin Park, Young-Pyo Jeon, Jin-Yong Hong, Ho-Seok Park, Jea-Uk Lee

**Affiliations:** 1 School of Chemical Engineering, Sungkyunkwan University (SKKU), 2066 Seobu-ro, Jangan-gu, Suwon 440-746, Korea; kyj0905@krict.re.kr; 2 Department of Advanced Materials Engineering for Information and Electronics, Kyung Hee University, 1732 Deogyeong-daero, Giheung-gu, Yongin-si 446-701, Korea; bin01@khu.ac.kr; 3 Carbon Industry Frontier Research Center, Korea Research Institute of Chemical Technology (KRICT) 141, Gajeong-ro, Yuseong-gu, Daejeon 305-600, Korea; ypjeon@krict.re.kr (Y.-P.J.); jyhong@krict.re.kr (J.-Y.H.)

**Keywords:** electronic device, thermal management system, polymer composites, carbon fillers, thermal conductivity

## Abstract

With the development of microelectronic devices having miniaturized and integrated electronic components, an efficient thermal management system with lightweight materials, which have outstanding thermal conductivity and processability, is becoming increasingly important. Recently, the use of polymer-based thermal management systems has attracted much interest due to the intrinsic excellent properties of the polymer, such as the high flexibility, low cost, electrical insulation, and excellent processability. However, most polymers possess low thermal conductivity, which limits the thermal management applications of them. To address the low thermal conduction of the polymer materials, many kinds of thermally conductive fillers have been studied, and the carbon-based polymer composite is regarded as one of the most promising materials for the thermal management of the electric and electronic devices. In addition, the next generation electronic devices require composite materials with various additional functions such as flexibility, low density, electrical insulation, and oriented heat conduction, as well as ultrahigh thermal conductivity. In this review, we introduce the latest papers on thermally conductive polymer composites based on carbon fillers with sophisticated structures to meet the above requirements. The topic of this review paper consists of the following four contents. First, we introduce the design of a continuous three-dimensional network structure of carbon fillers to reduce the thermal resistance between the filler–matrix interface and individual filler particles. Second, we discuss various methods of suppressing the electrical conductivity of carbon fillers in order to manufacture the polymer composites that meet both the electrical insulation and thermal conductivity. Third, we describe a strategy for the vertical alignment of carbon fillers to improve the through-plane thermal conductivity of the polymer composite. Finally, we briefly mention the durability of the thermal conductivity performance of the carbon-based composites. This review presents key technologies for a thermal management system of next-generation electronic devices.

## 1. Introduction

As microelectronic devices with the miniaturization and integration of electronic components have been developed, thermal management systems are getting more and more important. The considerable heat energy emitted from the dense packing of integrated circuits and electronic components can affect the performance and service life of microelectronic devices [1]. In the past, copper plates and aluminum pans were used to dissipate heat from conventional electronic products, such as large screen displays and desktop computers. However, these materials cannot be applied in microelectronic devices, which limit the weight and space inside them. Thus, to solve this problem, efficient thermal management systems should be designed with lightweight materials, which have outstanding thermal conductivity and processability.

**Figure 1:**
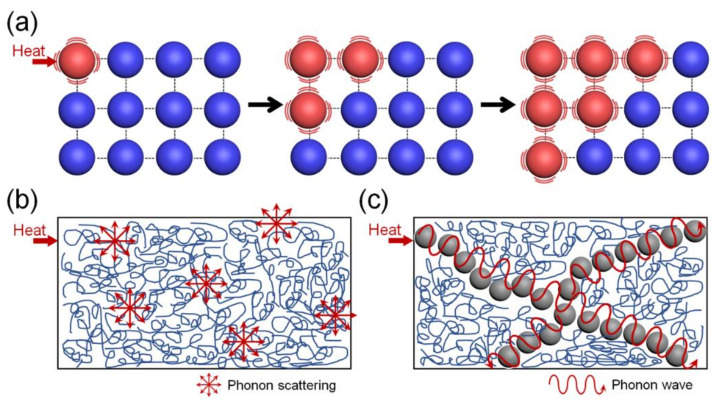
(**a**) Mechanism of thermal conduction in a crystalline material. Schematic diagrams of heat transfer in (**b**) a pure polymer and (**c**) a polymer filled with a thermally conductive filler.

Recently, the use of polymer-based thermal management systems has attracted much interest in the academic and industrial fields, in light of the high flexibility, low cost, electrical insulation, and excellent processability of them. However, the intrinsic amorphous arrangement and vibrations of the polymeric chains interfere with the thermal conduction of the polymer materials. In general, the heat transfer in conventional crystalline materials is realized via a lattice vibration mechanism, as shown in Figure 1a [2]. External thermal energy is transformed to vibration energy in the crystalline materials and transferred to the surrounding atoms. Unlike the pure crystalline materials, the conduction of thermal energy in a polymer can be achieved via a phonon transfer process, as shown in Figure 1b [3]. It has been reported that the phonon “transferring” through the phase interface depends on the vibration frequencies of the two phases [4]. However, the phonon “scattering” phenomenon is quite pronounced in polymer materials due to the vibrational frequency mismatches at the interfaces between impurities and lattice defects [5], which results in the low thermal conductivities, such as that of polymethylmethacrylate and polystyrene being about 0.2 W·m^−1^·K^−1^.

To address the low thermal conduction of the polymer materials, many studies have been carried out to incorporate thermally conductive fillers within the polymers, where the thermally conductive fillers connect to each other to form a continuous network structure in the polymer matrix (Figure 1c). Since the thermal energy can be directly transferred through this network structure with minimized phonon scattering, the inherent thermal conductivity, structure, and dispersity of the thermally conductive fillers are important factors to determine the overall thermal conductivity of the polymer composites. Until now, three kinds of thermally conductive fillers have been widely used: ceramic fillers such as boron nitride (thermal conductivity (*k*) values of 250–300 W·m^−1^·K^−1^), silicon carbide (*k* ~ 75–125 W·m^−1^·K^−1^), and beryllium oxide (*k* ~ 250 W·m^−1^·K^−1^) [6,7,8]; metallic fillers such as gold (*k* ~ 345 W·m^−1^·K^−1^), copper (*k* ~ 480 W·m^−1^·K^−1^), and aluminum (*k* ~ 200 W·m^−1^·K^−1^) [9,10,11]; and carbon fillers such as graphite (*k* ~ 300–500 W·m^−1^·K^−1^), carbon fiber (*k* ~ 2000 W·m^−1^·K^−1^ in the axial direction and *k* ~ 10–110 W·m^−1^·K^−1^ in the transverse direction), carbon nanotubes (*k* ~ 3000–6000 W·m^−1^·K^−1^), and graphene (*k* ~ 1800–6500 W·m^−1^·K^−1^) [12,13,14,15]. Among many candidate composite materials, carbon-based polymer composites have attracted intensive attention from both the academic and industrial fields due to the outstanding properties of carbon fillers, such as ultrahigh thermal conductivity, excellent electrical conductivity, mechanical robustness, flexibility, and a large surface area.

Previously, some papers reviewed thermally conductive polymer-based composites [16,17]. In particular, Kim et al. produced a review paper that could increase the comprehensive understanding of carbon-based polymer composites [4]. They summarized the physical factors affecting the thermal conductivity of polymer composites filled with carbon fillers, such as dispersion, filler size and shape, thermal percolation, and the synergistic incorporation of different types of fillers. In this review, unlike the previous review papers, the latest papers are introduced on highly conductive polymer composites, based on the carbon fillers with sophisticated structures for carrying out a specific performance.

**Figure 2:**
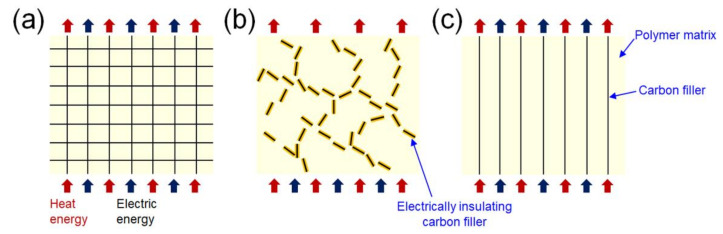
Schematics of thermal conductance in polymer composites with continuous 3D architectures of carbon fillers (**a**), electrically insulating carbon fillers (**b**), and vertically aligned carbon fillers (**c**).

The first topic is the preparation of high-performance polymer composites by constructing a continuous 3D architecture of the carbon fillers. The second topic is the development of the polymer composites with thermally conductive yet electrically insulating carbon fillers. The third topic is the development of polymer composites with vertically aligned carbon fillers for the enhancement of the through-plane thermal conductivity of the composites. The last topic is the durability of the thermal conductivity performance of carbon-based composites. The four topics introduced in this review have become a recent research trend in the field of thermally conductive polymer composites, and we expect that they will be key technologies for the thermal management of cutting-edge electronic devices in the future. A brief overview of the topics in this review is shown in Figure 2.

## 2. Polymer Composites with a Continuous 3D Architecture of Carbon Fillers

**Figure 3:**
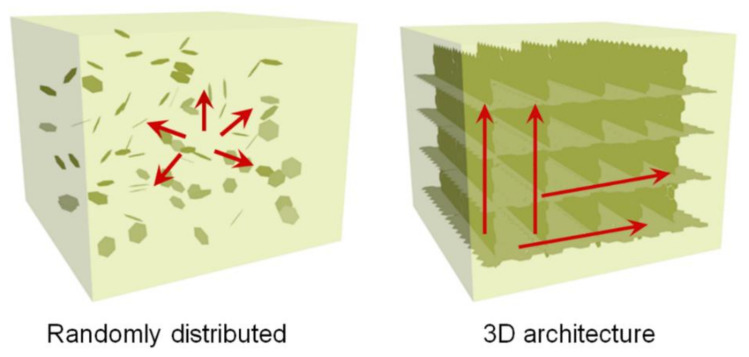
Schematic illustrations of thermal conductance in polymer composites with randomly distributed carbon fillers and a continuous 3D architecture of carbon fillers.

As was already mentioned, pure polymers have inferior thermal conductivity due to disordered polymer molecular chains and weak molecular interactions. Although attempts to improve the thermal conductivity of polymers by increasing crystallinity and maximizing the chain orientation have been reported [18,19], this approach has limitations in that it can only be applied to linear chain polymers with good crystallization. The incorporation of nanofillers with high thermal conductivity to the polymer matrix is a more convenient approach to enhance the performance of polymer composites. However, this method also has a problem in that the thermal conduction of polymer composites strongly depends on the properties of the nanofiller, such as morphologies, sizes, material types, surface properties, and especially dispersibility and intrinsic thermal conductivities. Furthermore, since heat is transferred by forming a percolation network between the nanofillers in a polymer matrix, high thermal conductivity can be achieved only when a relatively large amount of fillers are well dispersed, which greatly degrades the processability and applicability of the polymer composites. Therefore, the preconstruction of a continuous 3D connected network with the conductive fillers is an efficient strategy for the preparation of the thermally conductive polymer composites by reducing the thermal resistance at the filler–matrix interface and between individual filler particles, as schematically displayed in Figure 3. The thermal conductivity values of the recently published polymer composites with a continuous 3D architecture of carbon fillers are summarized in Table 1.

Graphene oxide (GO) is a graphene derivative with very good solubility and processability, produced by the chemical oxidation of graphite [26]. Yu et al. developed a 3D graphene network based on GO by the formation of graphene aerogels with both a conductive network and high porosity [20]. The graphene aerogels were obtained by freeze drying the molded GO pastes, and then the thermal conductivities of the graphene aerogels were improved by high temperature annealing at 2800 °C. Finally, the graphene aerogels had a density of ~0.042 g·cm^−3^ and a porosity of ~98.2% impregnated with 1-octandecanol, resulting in the preparation of highly thermally conductive composites (4.28 W·m^−1^·K^−1^). Although this process produced graphene aerogels having a light weight and high thermal conductivity by using highly processable GO, it had the difficulty of carrying out the high temperature heat treatment to remedy the defects of GO.

Yang et al. prepared hybrid aerogels consisting of GO and graphene nanoplatelets (GNP) to obtain composite phase change materials [22]. The hybrid aerogels were developed via freeze drying of a GO/GNP solution, which was then introduced into polyethylene glycol (PEG) via vacuum impregnation. In this hybrid structure, GO nanosheets built up the 3D framework to support the shape, and the GNP uniformly covered the network structure of GO to construct a thermally conductive pathway. The hybrid graphene/PEG composite recorded an improved thermal conductivity of 1.43 W·m^−1^·K^−1^ with 0.45 wt% GO and 1.8 wt% GNP. In addition, good structural stability and an energy conversion from light to heat were realized with the hybrid graphene/PEG composite. This method provides a simple and environmentally friendly way to achieve simultaneous enhancement of the thermal conductivity, energy storage density, and shape stabilization of the polymer composites.

Bai et al. prepared a composite by forming 3D graphene networks synthesized via the chemical vapor deposition (CVD) method and infiltrating it with carbon black (CB) and polydimethylsiloxane (PDMS) [23]. CB was well-dispersed not only in the space of the interconnected graphene network, but also in the interior of the network arms. A synergic effect between the continuous graphene network and well-dispersive CB significantly increased the thermal conductivity of 0.686 W·m^−1^·K^−1^. (The 8 wt% CB/GF/PDMS composite was 222% and 72% higher in thermal conductivity compared to the pure PDMS and GF/PDMS composite, respectively.) Moreover, the covalently bonded graphene network prevented molecular chains from moving, which improved the thermal stability of the composites. However, this approach has a disadvantage in that the preparation of the 3D graphene and the etching process are not cost effective. In addition, the thermal conductivity is still low, despite the complex preparation process.

**Figure 4:**
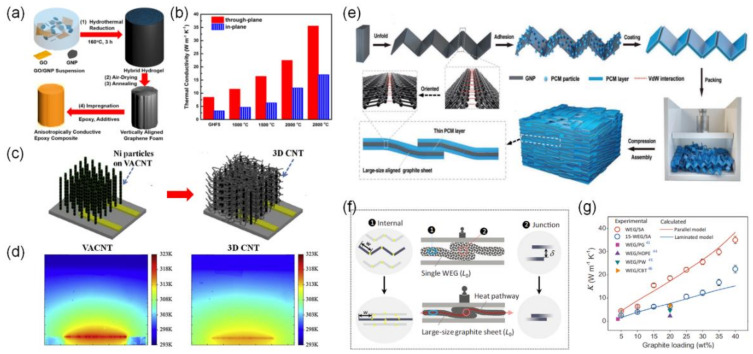
(**a**) Schematic illustration of the preparation of 3D graphene hydrogel composites and (**b**) the thermal conductivity of the composites after treatment at different annealing temperatures [23]. Copyright the American Chemical Society. (**c**) Preparation procedure of the secondary CNTs and (**d**) thermal maps of the VACNT and 3D CNT [24]. Copyright Elsevier. (**e**) Schematic diagram for synthesizing phase change composites. (**f**) The configuration diagram of the compressed worm-like expanded graphite (WEG). The red circles represent the thermal resistance junctions between the adjacent WEG (or graphite sheets). The blue ellipses represent the partial WEG (or graphite sheets). The red arrows represent the heat flow. The yellow, semitransparent solid circles represent the connections between GNPs. (**g**) Thermal conductivity of the WEG/SA and 15-WEG/SA composite blocks as a function of the graphite loading [25]. Copyright Wiley.

In his excellent review paper, Feng et al. explained that the most important factors determining the thermal conductivity of 3D graphene network-based composites are the quality and density of the graphene 3D network [5]. In order to manufacture composite materials that satisfy both requirements, Yu et al. designed graphene hybrid foams with high density by the hydrothermal reduction of GO in the presence of GNPs (Figure 4a) [24]. Similar to the previously introduced hybrid graphene network, rGO acted as an interconnected network during the hydrothermal reduction process, while the incorporated GNPs played an important role in making the thermal conduction network denser. In addition to this process, graphitization of the graphene hybrid network at 2800 °C improved the quality of the graphene foams (removing the residual oxygen-containing functional groups and healing the defects of their reduced graphene oxide component). The resultant hybrid rGO/GNP/epoxy composites exhibited an ultrahigh through-plane thermal conductivity of 35.5 W·m^−1^·K^−1^ at a graphene loading of 19.0 vol% (Figure 4b).

Unlike the graphene aerogel structure, one dimensional shaped CNTs can form CNT arrays oriented in one direction, which has a great effect on the directional heat transfer of CNT array-based composite materials [27]. Ivanov et al. prepared millimeter tall, vertically aligned CNT arrays by CVD synthesis and recorded their thermal conductivity values in air (15.3 W·m^−1^·K^−1^) and epoxy-infiltrated composites (5.5 W·m^−1^·K^−1^). It has been reported that the main factors to determine the thermal conductivities of the CNT array-based composites are as follows: the growth density and orientation of the CNT arrays and the defect levels and lengths of the individual CNTs composing the array [28].

Instead of aligned CNT arrays, Kong et al. used a 3D CNT network structure, which was prepared by cross-linking a vertically aligned CNT (VACNT) array with randomly oriented secondary CNTs grown directly from the walls of the primary VACNTs (Figure 4c) [24]. They compared various catalyst preparation methods to produce a dense network of secondary CNTs and chose the chemical impregnation method. The in-plane thermal conductivity of the obtained 3D CNT network was recorded as 5.40 W·m^−1^·K^−1^, much higher than that of the VACNT array (0.10 W·m^−1^·K^−1^), due to the extra thermal pathways provided by the secondary CNTs (Figure 4d). In the case of the 3D network structure, the interfacial thermal resistance should be minimized to improve the thermal conductance of the composites, and Kong et al. solved this problem by the direct growth of randomly oriented secondary CNTs from the walls of the primary VACNTs. However, this method requires several preparation steps: two-step CNT growth to form a 3D CNT structure and the removal of the Ni metal catalyst, which is unsuitable for the practical applications of the polymer composites due to the increased processing cost and complexity.

In order to reduce the expensive production cost of nanocarbons, such as graphene and CNTs, Wang et al. constructed a 3D network in the phase-change composite materials using expanded graphite (Figure 4e) [25]. They also pointed out the drawbacks of the nanocarbon fillers dispersed in the composites, namely random geometric contact and an interface thermal resistance bottleneck. To solve these problems, large-sized aligned graphite sheets were formed inside the phase change composites by mechanically compressing a worm-like expanded graphite with hydrophobic polymer coatings. The strong interactions between the millimeter-scale aligned graphite sheets derived by mechanical compression weakened the phonon scattering at the interfaces, which contributed to decreasing the interfacial thermal resistance and improving the thermal conduction of the composites (Figure 4f). Finally, the phase-change composites recorded a thermal conductivity as high as 35.0 W·m^−1^·K^−1^ at graphite loadings of 40.0 wt% (Figure 4g). The use of the expanded graphite and simple compression process can offer a promising route to low-cost and scalable applications of the phase-change composites for thermal energy storage and harvesting devices.

## 3. Polymer Composites with Thermally Conductive Yet Electrically Insulating Carbon Fillers

Although carbon fillers such as graphite, graphene, and CNT have ultra-high thermal conductivities [29,30,31], due to their high electrical conductivities, they have been limited in their use in various electronic parts, which should satisfy both thermal conductivity and electrical insulation at the same time [32]. Numerous techniques have been developed to suppress the electrical conductivities of the carbon materials while maintaining their intrinsic excellent thermal conductivities. In this part, we introduce several ways for developing thermally conductive yet electrically insulating carbon fillers, including the coating and hybridization of carbon materials with other nanomaterials, band gap tuning, and the morphological control of carbon fillers in polymer composites. Table 2 summarizes the thermal conductivities of previously reported polymer composites with electrically insulating carbon filler.

To prevent the formation of electrically conductive networks, carbon materials are usually coated with inorganic materials such as MgO [33], SiO_2_ [34], Al_2_O_3_ [35], and boron nitride (BN) [36]. Recently, Zhang et al. modified the surfaces of MgO nanoparticles and carbon fibers (CF) to prepare a thermally conductive but electrically insulating filler for a polymer matrix [33]. By treating these materials with the coupling agents, MgO-coated carbon fibers (CF-MgO) were synthesized, and CF-MgO/Nylon 6 composites were prepared by melt extrusion with a twin screw extruder. The thermal conductivity of the Nylon 6 composite with 20 wt% CF-MgO loading was 0.748 W·m^−1^·K^−1^, higher than that of the pure Nylon 6 (0.276 W·m^−1^·K^−1^) while maintaining the surface electrical resistivity. Although this approach is simple and can be applied to the various engineering polymers that are widely used in electronic parts, it may impair the original properties of the CF, owing to the presence of an acid treatment process for modifying the CF surface.

Subsequently covering the carbon surface with amphiphilic materials and then with a ceramic layer is a suitable way to insulate the carbon material while avoiding the formation of defects through a harsh surface modification process. For example, Shim et al. applied an amphiphilic substance, either Triton X-100 or PEG, on the graphite surface, and then silica was coated by a sol–gel method [34]. The composite using the silica-coated graphite fillers and thermoplastic polyester elastomer (TPEE) matrix exhibited a high thermal conductivity of 1.44 W·m^−1^·K^−1^ and maintained its electrical insulating property over 10^10^ Ω·sq^−1^ with a 30 vol% filler loading. Although the covering with organic substances could protect the carbon surfaces from defects, the additional covering process is cumbersome and may degrade the original properties of the carbon materials. Accordingly, research on coating the carbon surface with insulating inorganic materials without a surface modification process will be described.

**Figure 5:**
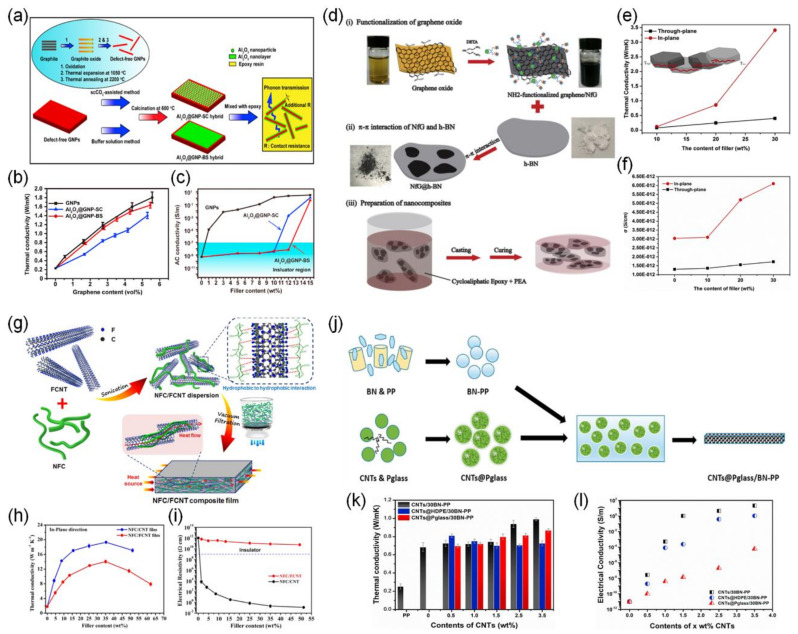
(**a**) Schematic illustration of the synthesis routes of Al_2_O_3_@GNP hybrids and their epoxy composites. (**b**) Comparison of electrical conductivities of epoxy composites filled with GNPs and their hybrids at 100 Hz. (**c**) Plots of thermal conductivity versus the GNP volume content of epoxy composites [35]. Copyright Elsevier. (**d**) Schematic illustration of the procedure to prepare the flexible composites. (**e**) Thermal and (**f**) electrical conductivities of CER/NfG@h-BN as a function of the filler content [36]. Copyright Elsevier. (**g**) Schematic illustration of the preparation of the NFC/FCNT composite films. (**h**) Thermal and (**i**) electrical conductivities of the NFC/FCNT and NFC/CNT composite films with different filler loadings [38]. Copyright the American Chemical Society. (**j**) Schematic illustration of the fabrication procedure for CNTs@Pglass/BN-PP. (**k**) Thermal and (**l**) electrical conductivities of CNTs@Pglass/30BN-PP as a function of the CNT content [40]. Copyright Elsevier.

Zhang and Yu et al. used electrically insulating Al_2_O_3_ to coat defect-free graphene nanoplatelets (GNP) without surface modification of the GNPs by using two different methods: (i) fast nucleation and hydrolysis of the Al(NO_3_)_3_ precursor with supercritical carbon dioxide (scCO_2_) and (ii) controlling the nucleation and hydrolysis of the Al_2_(SO_4_)_3_ precursor with a buffer solution (Figure 5a) [35]. The Al_2_O_3_@GNP hybrid, with the assistance of scCO_2_, was coated with homogeneous Al_2_O_3_ particles on the GNP surfaces, while the hybrid prepared with the help of a buffer solution was covered with a compact and flat Al_2_O_3_ layer. As a result, the buffer solution treatment brought about the Al_2_O_3_@GNP hybrid with superior thermal conductivity while retaining its electrical insulation. The epoxy composite exhibited a high thermal conductivity of 1.49 W·m^−1^·K^−1^ with a 12 wt% of the Al_2_O_3_@GNP hybrid filler loading, which was 677% higher than that of neat epoxy (Figure 5b,c). In this study, the thickness or amount of the coated inorganic insulating layer is an important factor in determining the thermal conductivity of a composite. However, Zhang and Yu et al. did not confirm the thermal conductivities of the composites with a controlled thickness or amount of the coating layer.

The thermally conductive but electrically insulating polymer composites were also obtained by the introduction of the hybrid materials of the electrically conductive carbon and insulating boron nitride. Zheng Su et al. mixed NH_2_-functionalized graphene (NfG) with hexagonal boron nitride (h-BN) to prepare NfG@h-BN hybrids (Figure 5d) [36]. The dispersion state and morphology of the NfG on the h-BN surface were controlled by adjusting the ratio of the NfG sheets and h-BN platelets. Adding a 30 wt% NfG@h-BN hybrid to the epoxy polymer significantly increased the thermal conductivity of the composite to 3.409 W·m^−1^·K^−1^, and maintained low electrical conductivity (Figure 5e,f). The low electrical conductivity was due to the separation of the NfG sheets on the surface of the h-BN and the inability to form a conductive network.

Recently, Feng et al. reported an easy way to prepare hybrid composite films with high in-plane thermal conductivity by using GO and BN microplates (BNMP) in the aqueous media [37]. The GO-BNMP composite films were prepared by simply pouring a homogeneous suspension of GO/BNMPs into a mold and drying without polymer resin. The π–π interactions between the aromatic regions of the GO sheets and the basal plane of the BNMPs induced the stable dispersion of the BNMPs in the GO suspension. Both the thermal conductivity and volume resistance of the GO-BNMPs composites enhanced as the content of BNMPs increased, and a maximum thermal conductivity of 10.3 W·m^−1^·K^−1^ was recorded at 50 wt% of BNMP loading. Moreover, they reported that the composite film showed outstanding mechanical flexibility and satisfactory electrical insulation. Compared with other methods, the hybrid process is easy to handle and can be extended to other research fields as well.

Band gap tuning is one of the smart approaches to develop electrically insulating carbon fillers for the preparation of thermally conductive polymer composites. Fluorination of carbon nanomaterials is an efficient method to tune their band gap via the structural transformation of the carbon–carbon bonds from sp^2^ to sp^3^ configuration [42]. For example, through the complete fluorination of graphene, an electrically insulating graphene sheet was prepared with a high band gap of ~3.8 eV.

Wu et al. prepared a free-standing composite film by vacuum filtration of a mixture of nanofibrillated celluloses (NFCs) and fluorinated CNT (FCNT) (Figure 5g) [38]. The fluorination of CNTs changed the intrinsic graphitic lattice of the CNTs and caused an irregular atomic arrangement, eventually decreasing the electrical conductivity. The resultant NFCs/FCNTs composite film represented a high thermal conductivity of 14.1 W·m^−1^·K^−1^ at 35 wt% FCNT and excellent electrical insulation of more than 10^10^ Ω·cm (Figure 5h,i). Moreover, these composite films showed excellent mechanical flexibility.

Recently, Kim et al. produced an exfoliated graphene fluoride (EGF) film by directly exfoliating a graphite fluoride into the graphene fluoride solution using a ball milling method and then vacuum-assisted filtration [39]. The thinner EGF film could minimize the phonon scattering between the adjacent sheets and facilitate the phonon transfer in the horizontal structure of the EGF sheets, and as a result, the 10 μm EGF film showed a high thermal conductivity of 242 W·m^−1^·K^−1^. However, the relationship between the thermal conductivity and electrical insulation characteristics of the carbon fillers, depending on the degree of fluorination, has not been verified, so further research is needed.

The selective distribution of carbon fillers in one polymer domain of the polymer blend system is a way to prevent the formation of electrically conductive networks in the whole polymer composites. Fu et al. mixed the CNT and phosphate glass (Pglass) to obtain CNTs@Pglass particles and then mixed them with polymer composites based on polypropylene (PP) containing BN to prepare CNT@Pglass/30BN-PP (Figure 5j) [40]. Due to the large interfacial tension between the Pglass and the polymer matrix, CNTs were isolated in the Pglass domain and could not form a CNT network. On the other hand, BN contributed to the formation of a thermal conduction network by connecting the CNTs@Pglass particles. The CNTs@Pglass/30BN-PP composite with 3.5 wt% CNT loading exhibited a thermal conductivity of 0.87 W·m^−1^·K^−1^ and maintained good electrical insulation properties (Figure 5k,l).

Similarly, Morishita et al. prepared morphologically controlled multiwalled CNT (MWCNT)/polyamide-6 (PA6)/poly(*p*-phenylene sulfide) (PPS) composites, having enhanced thermal conductivity and electrical insulation [41]. They explained that the ends of the MWCNTs were capped with PPS nanodomains to block the conductive network, and the MWCNT-PPS nanodomains were homogeneously dispersed in the polyamide-6 matrix. Although these composite materials were able to increase the elastic modulus, high thermal conductivity was not obtained (0.435 W·m^−1^·K^−1^) due to the low MWCNT content (1 vol%).

The recently proposed approaches have focused on the morphological control of the carbon filler in polymer composites, where the carbon filler is trapped by another polymer (e.g., Pglass) rather than the matrix. Therefore, a continuous network of conductive fillers cannot be formed, and the interfaces between the heterogeneous materials increase considerably, which could degrade the thermal conductivity of the composites. To solve these problems, interfacial thermal resistance should be minimized, or a new structural design is required.

## 4. Polymer Composites with Vertically Aligned Carbon Fillers

The third topic of this paper is the study of vertical alignment of the carbon fillers to improve the through-plane thermal conductivity of the composite materials. In general, the thermally conductive polymer/carbon composites in the film form have much lower through-plane thermal conductivities (0.5–10 W·m^−1^·K^−1^) than the in-plane thermal conductivities (10–100 W·m^−1^·K^−1^). Recently, many studies on the vertical alignment of carbon fillers using various processing techniques have been reported, since high through-plane thermal conductivity is increasingly required in the field of electronic parts. Table 3 summarizes the thermal conductivity values of the previously reported polymer composites with vertically aligned carbon fillers.

Ma et al. used electrostatic flocking to make vertically aligned carbon fiber arrays on a planar substrate [43]. When applying a potential of −30 kV, a pile of rigid and long (~1 mm in length and 10 µm in diameter) carbon fibers, placed on one face of two parallel electrodes, was charged and propelled toward the opposing electrode. By pouring the fluorinated rubber onto the vertically aligned carbon fiber arrays, a highly uniform composite was formed. The composite showed both high through- and in-plane thermal conductivities of 23.3 and 8.96 W·m^−1^·K^−1^, respectively, with a carbon fiber filler loading of 13.2 wt%. However, this approach still has a lot of room for performance improvement, since the density level, degree of alignment, ratio of CF that spans from bottom to top, and the filling fraction have not been optimized.

**Figure 6:**
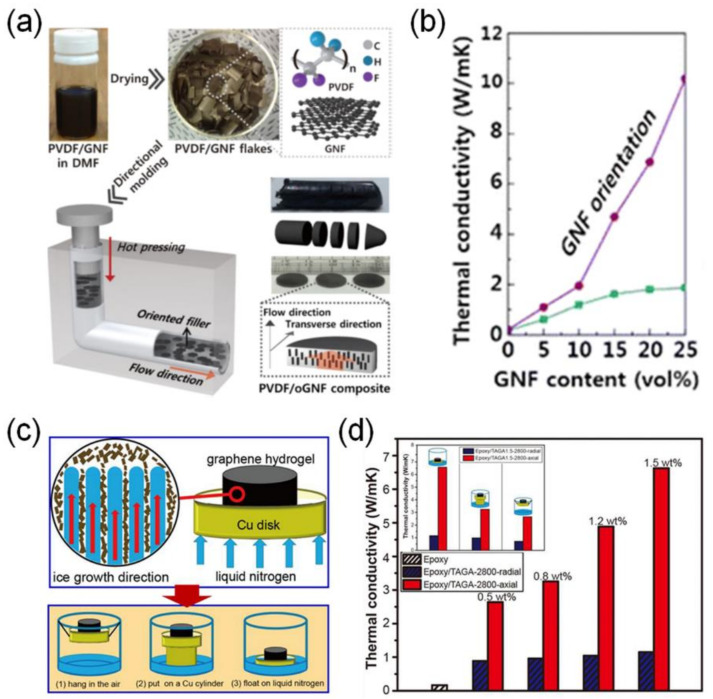
(**a**) Schematic illustration of the fabrication process of GNFs preferentially aligned in PVDF through an L-shaped kinked tube. (**b**) Thermal conductivity of PVDF/GNF and PVDF/GNF composites with different GNF amounts [44]. Copyright the American Chemical Society. (**c**) Schematic illustration of the fabrication of vertically aligned graphene aerogels. (**d**) Thermal conductivity of the vertically aligned graphene aerogel composite [49]. Copyright Elsevier.

Park et al. fabricated polymer composites with high directional thermal conductivity by controlling the orientation of the graphene nanoflake (GNF) fillers [44]. They pointed out the need for the fabrication of polymer composites with a high through-plane thermal conductivity but low filler loading. Therefore, melt compression of poly(vinylidene fluoride) (PVDF) and GNF films was performed in an L-shape kinked tube to yield a polymer composite with preferentially aligned GNF fillers perpendicular to the melt flow direction (Figure 6a). The PVDF/GNF composite exhibited a through-plane thermal conductivity of 10.19 W·m^−1^·K^−1^ with 25 vol% GNF loading (Figure 6b). Although this method derived high through-plane thermal conductivities with a small amount of filler loading, application to the composite materials with various shapes and sizes is limited, because it requires a melt compression process through an L-shape kinked tube. Thereafter, many research groups manufactured the vertically aligned graphene fillers via various methods, such as rolling up the graphene film manually [45], 3D printing of the polymer/graphite flake composite filament [46], and arranging the graphene film followed by infiltration with epoxy resin [47].

Recently, some approaches on manufacturing a composite, which satisfy both the vertical alignment and interconnected network through the directional freeze drying of graphene, have been reported. Wong et al. constructed vertically aligned and interconnected graphene networks via three-steps, including the formation of GO liquid crystals, oriented freeze casting, and thermal reduction of the aligned GO [48]. After infiltration with epoxy resin in the vertically aligned graphene networks, the resultant composite showed a through-plane thermal conductivity of 2.13 W·m^−1^·K^−1^ with a very low graphene loading of 0.92 vol%.

Yu et al. also manufactured the fully graphitized graphene aerogels with a vertically aligned graphene network (Figure 6c) [49]. As a starting precursor, GO hydrogels were prepared by directional freezing, and subsequent freeze drying and graphitization at 2800 °C resulted in vertically aligned and networked graphene aerogels. The final epoxy composite recorded a through-plane thermal conductivity of 6.57 W·m^−1^·K^−1^ with 1.5 wt% (~0.75 vol%) graphene aerogel fillers (Figure 6d). These polymer composites with vertically aligned and ultralight graphene aerogels are expected to be thermal management parts for next-generation flexible electronic devices [50], because they can impart excellent mechanical properties (storage modulus, elastic modulus, and compressive strength) as well as a high through-plane thermal conductivity with a very small amount of carbon filler loading.

## 5. Stability of the Thermal Conductivity Performance of Polymer Composites

The stability of the thermal conductivity performance is an important factor that must be considered to design polymer composites for the heat dissipation applications. Xiao et al. achieved not only a high thermal conductivity, but also durability of the carbon-based polymer composites by fabricating a carbon fiber foam with a mixture of cellulose and carbon fiber, using the ice template method and then compounding it with PDMS [51]. When loaded with 12.8 vol% of the filler, the polymer composite showed a thermal conductivity of 6.04 W·m^−1^·K^−1^, and as a result of repeated heating and cooling cycle tests between 25 °C and 125 °C, it exhibited stable heat dissipation performance without significant degradation. Zhiduo et al. prepared graphene foam using polyurethane foam as a template and then compounded it by injecting the epoxy resin [52]. The recorded thermal conductivity of the composite was 8.04 W·m^−1^·K^−1^ with a filler loading of 6.8 wt%. They also carried out the durability test by repeating 15 heating and cooling cycles between 25 °C and 100 °C. The carbon-based polymer composite with 6.8 wt% filler loading showed small deviations, which suggested stable heat conduction ability after the long-term operation of the device.

## 6. Conclusions and Future Outlooks

In this review, we introduced novel research papers on thermally conductive polymer composites based on various carbon fillers, such as CNT, graphene, graphite, and carbon fibers. In order to solve the heat generation problem of electronic devices, many research papers have already been published on polymer composite materials. In this paper, we focused on the composite research with sophisticated structures that meet the new properties, such as the flexibility, low density, electrical insulation, and oriented heat conduction required by next-generation electronic devices. We classified new composite materials into three categories: polymer composites with (i) three-dimensional networked architecture of the carbon fillers, (ii) thermally conductive yet electrically insulating carbon fillers, and (iii) vertically aligned carbon fillers.

1. The construction of a continuous 3D networked structure with carbon fillers is a promising approach for the fabrication of thermally conductive polymer composites with a small amount of filler loading. To obtain the 3D networked carbon structures, graphene aerogels, CNT arrays, and expanded graphite have been developed. Many scientists mentioned that the most important factors for determining the thermal conductivity of a 3D carbon network-based composites are the quality and density of the carbon 3D network. To satisfy both requirements, new approaches for the hydrothermal reduction of 3D carbon frameworks in the presence of the thermally conductive carbon fillers in powder form have been proposed;
2. Although nanocarbon fillers have ultrahigh thermal conductivity, they have been limited in use on various electronic parts because of their high electrical conductivity. We introduced some papers on the development of thermally conductive yet electrically insulating carbon fillers, including the coating and hybridization of carbon materials with other nanomaterials, band gap tuning, and the morphological control of carbon fillers in polymer composites. In general, nanocarbon fillers have been coated and hybridized with inorganic materials such as MgO, SiO_2_, Al_2_O_3_, and BN to prevent the formation of electrically conductive networks. Through these approaches, thermally conductive composite materials can be applied to various electronic parts requiring electrical insulation, such as electronic circuit boards;
3. Some electronic parts request heat dissipation in the thickness direction rather than the surface direction, depending on the working mechanism and structure of the electronic product. However, in general, the thermally conductive polymer composites in the film formation have much lower through-plane thermal conductivities than the in-plane thermal conductivities. Therefore, we introduced research on the vertical alignment of carbon fillers in the polymer matrix. Although these studies have recently been started, some composite materials with high through-plane thermal conductivities were developed through various processing techniques, such as electrostatic flocking, 3D printing, and directional freeze drying of the nanocarbons;

Despite the intensive efforts of many researchers, there are still many problems to be solved in order for the research of polymer composites to move from academic research to industrial applications. First, a mass production method for the formation of the 3D architecture and vertical alignment of carbon fillers has never been developed. It requires a novel approach that should be cost-effective and simpler than the previously published methods. Second, we need to find a way to minimize the thermal interface resistance between the carbon fillers and polymer matrix. Finally, it is necessary to develop an optimal method for preparing a thermally conductive yet electrically insulating carbon filler. In addition, research on the structural control and alignment of the electrically insulating carbon filler should be continued for the application of the carbon-based polymer composites to the thermal management of cutting-edge electronic devices in the future.

## Figures and Tables

**Table 1:** Thermal conductivity (*k*) values of the various polymer composites with a continuous 3D architecture of carbon fillers.

Fillers	Matrix	Filler Loading	*k* (W·m^−1^·K^−1^)	Enhancement in *k* (%)	References
Graphen eaerogel	1-octadecanol	5.0 wt%	4.28	94.5%	[20]
GO/GNP hybrid aerogel	PEG	0.45/1.8 wt%	1.43	361%	[21]
CVD graphene network with CB	PDMS	8 wt%	0.686	222%	[22]
GO/GNP hybrid foam	Epoxy	19.0 vol%	35.5	884%	[23]
3D CNT network	-	-	5.40	-	[24]
Expended graphite	Stearic acid	40.0 wt%	35.0	-	[25]

**Table 2:** Thermal conductivity (*k*) values of various polymer composites with electrically insulating carbon fillers.

Fillers	Matrix	Filler Loading	*k* (W·m^−1^·K^−1^)	Enhancement in *k* (%)	Refs
CF-MgO	Nylon 6	20 wt%	0.748	-	[33]
silica@graphite	TPEE	30 vol%	1.44	-	[34]
Al_2_O_3_@GNP	epoxy	12 wt%	1.49	667%	[35]
NfG@h-BN	epoxy	30 wt%	3.409	-	[36]
GO	BN	50 wt%	10.3	-	[37]
FCNT	NFCs	35 wt%	14.1	729%	[38]
EGF	-	-	242	-	[39]
CNTs@Pglass/BN	PP	3.5 wt%	0.87	-	[40]
MWCNT	PA6	1 vol%	0.433	-	[41]

**Table 3:** Thermal conductivity (*k*) values of various polymer composites with vertically aligned carbon fillers.

Fillers	Matrix	Filler Loading	*k* (W·m^−1^·K^−1^)	Enhancement in *k* (%)	References
CF	Rubber	13.2 wt%	8.9	10,986%	[43]
GNF	PVDF	25 vol%	10.19	-	[44]
GO	Epoxy	15.79 wt%	2.645	887%	[45]
Graphite	PA6/POE-g-MAH/PS	10 wt%	5.5	-	[46]
Graphene	Epoxy	44 vol%	384.9	3570%	[47]
GO	Epoxy	0.92 vol%	2.13	1231%	[48]
GO	Epoxy	1.5 wt%	6.57	5890%	[49]

## Data Availability

The data presented in this study are available on request from the corresponding author.
